# Influence of Climate on Phthisis

**Published:** 1862-05

**Authors:** 


### INFLUENCE OF CLIMATE ON PHTHISIS.

We find the following statement in the *Medical Times and Gazette*, which did it not betray a great want of knowledge on a subject which deeply interests the welfare of thousands, would be really amusing:

"The Empress of Austria, who, as your readers are aware, went some time ago to Corfu, under the care of Professor Skoda, for tuberculosis of the lungs, is reported considerably improved. The heat of Corfu is at present very great, amounting to 95° F. at mid-day, to 77° in the evening, and only a little less in the morning. Sudden changes of temperature, however, never take place, and even after sunset walking in the open air is not injurious to the patient. The climate of Corfu agrees remarkably well with the Empress, and the infiltration of the lungs, which had made alarming stride of late, has become stationary. The cough has quite ceased, and the general health of the illustrious patient is much improved. It has not yet been determined where the Empress will pass the winter, but it is settled that she leaves Corfu on its approach, as the winter there is not suitable for delicate patients suffering from diseases of the chest."

If we are to consider the climate of Corfu favorable for phthisical patients, where "walking in the open air after sunset is not injurious to the patient," we hardly know how high we ought to rate the climate of California, where not only can our patients walk in the open air after sunset without injury, but where they can sleep in the open air during six months in the year, with manifest advantage. During the past summer, we have had phthisical patients camping out in the mountains, and sleeping under the trees, in whom not only has the infiltration of the lungs become stationary, but in whom it has entirely disappeared.

Whilst on the subject of phthisis, we will endeavor to give as literal a translation as possible of a communication by Mr. Piorry, presented to the Academy of Medicine in Paris, furnishing, as it does, a sufficient reason why phthisis should still prove itself incurable to physicians of the older school.

"M. Piorry read a communication on the treatment of pulmonary phthisis. The author enumerated the long list of remedies that had been brought forward at different times against tuberculosis, and endeavored to prove that the use of the greater part of these remedies and therapeutical methods was founded either on a blind empericism, or on hypothetical theories. He observed that all the means of treatment had been finally condemned by clinical experience, and he believed that the only rational treatment of pulmonary phthisis, is that which reposes on the precise indications furnished by the exact or plessimetric diagnosis of monorganisms, and which, prescribing all the pretended specifics, combats each lesion, local or general, by the treatment it requires. M. Piorry insisted more particularly on the necessity of favoring expectoration, and thus preventing the *anoxemia* which migh result from the retention of muo-purulent secretions in the acrea-tree, on combatting the *splenemogolia* by sulphate of quinine, and the *chloro-anemia* by ferruginous medicines."

Some twenty years ago, we listened with both pleasure and profit to M. Piorry's lectures on Diagnosis, and we only regret that, at least as regards pulmonary consumption, the only progress he appears to have made since that time, has been in the invention of a barbarous and unintelligible nomenclature. As well might we treat gout by applying a cooling lotion to the great toe, as to base our treatment of phthisis on expectorants and other drugs. Behind the pulmonary disease exists the tuberculosis of the system, which must itself be remedied, if ever we hope to treat phthisis successfully.

We have already pointed out, in former numbers of this Journal, how this is to be effected—it certainly can never be accomplished by drugs.—*Pacific Med. & Surg. Journal.*

